# Lack of a protective effect of cotton dust on risk of lung cancer: evidence from two population-based case-control studies

**DOI:** 10.1186/s12885-015-1206-0

**Published:** 2015-04-02

**Authors:** Krista Yorita Christensen, Jérôme Lavoué, Marie-Claude Rousseau, Jack Siemiatycki

**Affiliations:** 1 Environmental Epidemiology and Population Health Research Group, University of Montreal Hospital Research Center (CRCHUM), Tour Saint-Antoine, 850 St. Denis Street, Montreal, QC H2X 0A9 Canada; 2 Department of Environmental and Occupational Health, University of Montreal, Montreal, QC Canada; 3 Department of Social and Preventive Medicine, University of Montreal, Montreal, QC Canada; 4 INRS − Institut Armand-Frappier, Laval, QC Canada

**Keywords:** Cotton dust, Wool dust, Lung neoplasms, Occupational exposure, Case-control studies

## Abstract

**Background:**

Lung cancer is the leading cause of cancer death in North America. Exposure to cotton dust has previously been reported to decrease the risk of lung cancer.

**Methods:**

We used data from two large case-control studies conducted in Montreal from 1979-1986 (Study 1) and 1996-2002 (Study 2) respectively, to examine the association between occupational exposure to cotton dust and risk of lung cancer. Cases were diagnosed with incident histologically-confirmed lung cancer (857 in Study 1, 1203 in Study 2). Population controls were randomly selected from electoral lists and frequency-matched to cases by age and sex (533 in Study 1, 1513 in Study 2). Interviews for the two studies used a virtually identical questionnaire to obtain lifetime occupational and smoking history, and several lifestyle covariates. Each participant’s lifetime occupational history was reviewed by experts to assess exposure to a number of occupational agents, including cotton dust. Odds ratios (ORs) and 95% confidence intervals (CIs) were estimated by unconditional logistic regression, adjusting for potential confounders.

**Results:**

The lifetime prevalence of exposure to cotton dust was approximately 10%-15% in both studies combined, with some variation by study and by sex. Overall there was no decreased risk of lung cancer among subjects exposed to cotton dust. Rather, among all subjects there was a suggestion of slightly increased risk associated with any lifetime exposure to cotton dust (OR = 1.2, 95% CI: 1.0-1.5). This risk appeared to be concentrated among cases of adenocarcinoma (OR = 1.6, 95% CI: 1.2-2.2), and among moderate and heavy smokers (OR = 1.3, 95% CI: 1.0-1.7). There was no association when restricting to cases of either squamous cell or small cell cancer, or among never smokers and light smokers. An analogous examination of subjects exposed to wool dust revealed neither increased nor decreased risks of lung cancer.

**Conclusions:**

There was no evidence that cotton dust exposure decreased risks of lung cancer.

## Background

Lung cancer is the leading cause of cancer death in North America, accounting for about a quarter of all cancer deaths [1,2]. Due to a lack of effective screening, most cases of lung cancer are diagnosed at a relatively advanced stage, and consequently survival is very low (15% five-year survival rate) [3]. Lung cancer likely results from a combination of genetic and environmental factors, including smoking and occupational exposures.

Many occupational exposures, including asbestos, silica, nickel, and hexavalent chromium, have been identified as lung carcinogens [4]. Cotton dust as an occupational exposure has been associated with adverse respiratory effects including byssinosis and diminished lung function [5]. Peculiarly, cotton dust exposure has also been linked with a decreased risk of lung cancer [6-10]. An early report of decreased lung cancer risk among cotton textile workers came from the United States, where a standardized lung cancer mortality ratio of 0.55 (95% CI: 0.39-0.76) was reported in Georgia [7]. Subsequently there have been some other reports of decreased lung cancer risk in cotton-exposed workers in North Carolina [9], China [11,12], the UK [8], and Poland [13]. In some of these studies the decreased risk was restricted to certain sex, smoking subgroups, or calendar years [8,9,13], and some of the decreased risks were not statistically significant [11]. Furthermore, there have been other reports from Australia [14], Lithuania [15], and Italy [16] which found no evidence of decreased risks. A 2009 meta-analysis of 11 studies reported a summary relative risk of lung cancer among textile workers of 0.71 (95% CI: 0.52-0.95), albeit with considerable variability between studies and equivocal dose-response information within studies [17].

This ostensible decreased risk is hypothesized to result from exposure to endotoxins contained in cotton dust. Endotoxins are components of Gram negative bacteria consisting of three components (O-specific polysaccharide, core polysaccharide, and lipid A), one of which (lipid A) appears to have anti-carcinogenic activity [18,19]. Further epidemiologic evidence for this hypothesis came from a study among female textile workers in Shanghai, in which cumulative exposure to endotoxin was associated with a significantly decreased risk of lung cancer, with a dose-response relationship observed (HR of 0.60 [95% CI: 0.43-0.83] for highest levels of exposure compared to no exposure) [6].

While there are some indications of biologic plausibility of a protective effect of cotton dust on lung cancer supported by some, albeit inconsistent, epidemiologic evidence, it is important to produce further complementary evidence to assess this hypothesis. Montreal, Canada, with a population of about 3 million, is a propitious locale for such analyses, with approximately 25,000 jobs in the textile and clothing industries, and 1000 companies in the metropolitan Montreal area.

We carried out two large case-control studies in Montreal to determine the association between a large number of occupational exposures, including various textile dusts, and cancer, with detailed data collected on smoking history and other potential confounders. We used this database to analyze the association between cotton dust and risk of lung cancer. While our primary interest was to assess a possible protective association with cotton dust exposure, we also analyzed wool dust and compared both sets of results because wool is an organic fiber of similar exposure prevalence to cotton, levels of contamination with endotoxins are much lower in wool than cotton dust, and endotoxin exposure among workers in wool processing is generally lower than in cotton processing [20]. If there were a general protective effect associated with working in the textile industry, it should manifest in reduced risks for both wool dust and cotton dust. The analysis of wool dust thus informs us about the specificity of any effect we might observe for cotton dust.

## Methods

### Design and study subjects

Both studies used a case-control design, with eligible subjects restricted to Canadian citizens resident in the Montreal area. Study 1, conducted from 1979 to 1986, included males aged 35 to 70 years diagnosed with cancer at any of 19 sites, including the lung. Study 2, conducted from 1996 to 2002, included men and women aged 35 to 75 diagnosed with a lung malignancy. In both studies, cases were ascertained in the 18 largest hospitals located in the metropolitan Montreal area; only incident, histologically confirmed cancers were included. In both studies, population controls were randomly sampled from population based electoral lists, stratified by sex and age to the distribution of cases. In Quebec, Canada, electoral lists were maintained by means of active enumeration of households until 1994; they are since then continually updated and are thought to represent nearly complete listings of Canadian citizens residing in the province. Ethical approval was obtained for each study from each participating hospital and academic institution (Institut Armand-Frappier, McGill University, Université de Montréal, Centre de recherche de l’Université de Montréal). All participating subjects provided informed consent. Additional details of subject ascertainment and data collection have been published previously [21-24].

In Study 1, 1082 lung cancer cases and 740 eligible population controls were identified and attempts were made to interview them. Of these, 857 (79%) cases and 533 (72%) controls completed the interview. Since Study 1 included cancers at several different sites, it was possible to constitute an additional control group for the lung cancer series, namely subjects with cancers at other sites. We refer to these as ‘cancer controls’. Sampling of these cancer controls was carried out excluding sites of the respiratory system; further, we subsampled the rest to ensure that none of the sites comprising the cancer controls would constitute more than 20% of the total. With these restrictions, the cancer control series consisted of 1349 subjects. In Study 2, there were 1203 cases (response rate 84%) and 1513 population controls (response rate 69%) interviewed. For subjects who were deceased or too ill to respond, we accepted proxy response from close family members; proxy response accounted for 23% of respondents in Study 1 (29% among cases and 13% among controls) and 21% in Study 2 (38% among cases and 8% among controls).

### Data collection

Data collection techniques and the variables ascertained were almost identical between Study 1 and Study 2. Interviews were divided into two parts: a structured section requested information on socio-demographic and lifestyle characteristics, and a semi-structured section elicited a detailed description of each job held by the subject in his working lifetime. Among the socio-demographic and lifestyle factors assessed were: ethnicity, socio-economic status as measured by education level, familial financial situation during childhood and current income, residential history, smoking history (smoking status, ages at initiation and cessation, periods of interruption, average number of cigarettes smoked per day over the lifetime), alcohol and coffee consumption, selected dietary factors, selected medical history conditions, household heating and cooking practices, and many others. Male subjects (Studies 1 and 2 combined) and female subjects (Study 2) had held a median of 4.0 jobs each. For each job held, a trained interviewer asked the subject about the company, its products, the nature of the worksite, the subject’s main and subsidiary tasks, and any additional information (e.g., equipment maintenance, use of protective equipment, activities of coworkers) that could provide clues about work exposures and their intensity. Occupations were coded according to the Canadian Classification and Dictionary of Occupations [25] and the Canadian Standard Industrial Classification [26,27]. For some occupations, supplementary questionnaires were used to assist interviewers with detailed technical probing [28]. A team of chemists and industrial hygienists examined each completed questionnaire and translated each job into a list of potential exposures using a checklist of 294 agents that included cotton dust, wool dust and several recognized lung carcinogens [23]. Endotoxin exposure was not on the checklist and its possible presence is only inferred from the presence of cotton dust.

In the two studies combined, nearly 30,000 jobs were evaluated. The team of coders spent about 50 person-years on these projects, including helping to develop the methodology, monitoring the quality of the interviewing, conducting background research on exposures in different occupations, coding the individual participants’ files, and recoding after the initial complete rounds of coding were finished. The final exposure codes attributed to a subject were based on consensus among the coders. Coders did not know the subject’s case or control status. For each substance considered present in each job, the coders noted three dimensions of information, each on a three-point scale: their degree of confidence that the exposure had actually occurred (possible, probable, definite), the frequency of exposure in a normal workweek (low [<5% of hours worked], medium [5% to 30% of hours worked], high [>30% of hours worked]), and the relative level of concentration of the agent (low, medium, high). Concentration levels were established with reference to certain benchmark occupations in which the substance is found. Specifically, we identified some hypothetical workplace situations *a priori* which would correspond to low, medium and high exposure for each substance, and the experts rated each real job against these benchmarks. Unfortunately, it proved impossible to reliably estimate absolute concentration values corresponding to the relative levels coded. Non-exposure was interpreted as exposure up to the level that can be found in the general environment. The exposure assessment was based not only on the worker’s occupation and industry, but also on individual characteristics of the workplace and tasks as reported by the subject; an illustrative example is in the Appendix of Parent et al [29].

### Statistical analysis

The main purpose for this analysis was to estimate the relative risk of lung cancer in relation to cotton dust and wool dust exposure. The availability of two studies, with two control groups among males in Study 1 and two sexes in Study 2, provided various opportunities. We first carried out analyses of the Study 1 data by comparing the cases separately with population controls and with cancer controls, defined above. There are pros and cons with cancer controls and population controls and we cannot affirm that one is necessarily more valid than the other [24,30]. Our prior belief was that the two control groups in Study 1 were equally valid. Consequently, to avoid giving greater weight to the more numerous cancer controls, we carried out a weighted logistic regression analysis giving equal weight to the two control series. For Study 2, we analyzed males and females separately. In order to maximize precision of estimates, we also conducted analyses pooling the Study 1 and Study 2 samples, both cases and controls, but only using population controls from Study 1 and Study 2. We thus present six distinct risk estimates: Study 1 using population controls among males, Study 1 using cancer controls among males, Study 1 with weighted population and cancer controls, Study 2 using population controls among males, Study 2 using population controls among females, and Study 1 plus Study 2 pooled using population controls among males plus females.

For each job in which the subject was exposed to cotton dust, we had the duration of the exposure in years and a set of ordinal values for confidence, frequency, and concentration. If a subject was exposed in two or more jobs, then lifetime values of confidence, frequency, and concentration were calculated by taking averages, weighted by the durations of the various jobs in which exposure occurred. The combination of duration, confidence, frequency, and concentration was used to categorize the lifetime exposure into categories as follows: unexposed, exposed at non-substantial level, exposed at substantial level. Because of latency considerations, exposures occurring within 5 years of diagnosis or interview were excluded. In order to be classified as exposed at the substantial level, a subject had to have been exposed at confidence of probable or definite, concentration and frequency of medium or high, and for duration greater than 5 years. All other exposed subjects were then classified in the non-substantial category. We consider this non-substantial/substantial dichotomy to be a simple proxy for cumulative exposure. The reference group for analyses consisted of those subjects who were never exposed to cotton dust. Wool dust was treated the same way.

Unconditional logistic regression was used to estimate odds ratios (ORs) and corresponding 95% confidence intervals (CIs). In order to control for the effect of potential confounders, multivariate models were constructed including the following covariates: age (continuous), ethnicity (French Canadian, other), years of education (0-7, 8-12, ≥13), familial financial situation during childhood (difficult, intermediate, comfortable), respondent status (proxy, self), smoking history (CSI, continuous), and ever exposure to some known occupational lung carcinogens - asbestos, chromium compounds, nickel compounds and silica. These occupational covariates were selected for inclusion because they are on the IARC Group 1 list of lung carcinogens [4], and because the prevalence of exposure to these substances in the study population was over 3%. Smoking history was parameterized using a comprehensive smoking index (CSI) as described in Leffondre et al [31]. The CSI takes into account the lifetime average number of cigarettes smoked per day, the total duration of smoking, and time since quitting in a single parameter index. It was demonstrated to provide a good fit to the data while maintaining a parsimonious representation of lifetime smoking history, in contrast to multivariable modelling of separate effects of several dimensions of smoking behavior [31]. We have previously described smoking characteristics of cases and controls from Study 2 according to quartiles of the CSI variable distribution [32].

For pooled analyses, we analyzed all lung cancer cases and population controls, and in addition to the covariates above, all models included Study (1 or 2) as an adjustment factor, since case/control ratios differed by study. Further, a series of analyses was conducted among self-respondents only. In addition, we also examined job and industry titles associated with exposure to cotton dust, and potential effect modification by smoking history and sex. For stratified analyses, never smokers were grouped with low smokers, defined as individuals having a CSI value at or below the 25^th^ percentile. Medium to heavy smokers were those with a CSI value above the 25^th^ percentile.

## Results

Demographic characteristics of the study populations are outlined in Table 1. Among the 857 lung cancer cases in Study 1 were 41.9% squamous cell carcinoma, 18.6% small cell carcinoma, and 19.5% adenocarcinoma. In Study 2, there were 1203 lung cancer cases: 29.3% squamous cell carcinoma, 17.2% small cell carcinoma, and 38.1% adenocarcinoma. Study 1 was restricted to males, while Study 2 included both males (60.3%) and females (39.7%). The age distribution was similar across all groups. In both studies, most participants were French Canadian, and most had less than 13 years of schooling. Nearly all the cancer cases were smokers, as well as a majority of male controls. About half of the females in Study 2 had ever smoked regularly. Among smokers, the majority smoked for over 30 years prior to interview. Except for histological subtypes, all of the covariates in Table 1 were included in multivariate estimates of odds ratios.

**Table 1:** **Selected demographic characteristics of the study population in two case-control studies, Montreal, Canada**

Characteristic (%)	Study 1			Study 2			
	Males			Males		Females	
	Cases	Population Controls	Cancer Controls	Cases	Controls	Cases	Controls
	(N=857)	(N=533)	(N=1349)	(N=738)	(N=899)	(N=465)	(N=614)
	%	%	%	%	%	%	%
Age group							
<55 years	22.8	25.1	29.4	13.5	11.7	23.2	24.9
55-64 years	50.3	42.6	42.1	32.7	28.6	35.1	31.1
65+ years	27.0	32.3	28.6	53.8	59.7	41.7	44.0
Respondent							
Self	70.6	87.4	80.8	60.0	90.1	66.0	95.3
Proxy	29.4	12.6	19.2	40.0	9.9	34.0	4.7
Ethnicity							
French	69.1	64.2	58.0	77.5	64.4	78.5	68.6
Other	30.9	35.8	42.0	22.5	35.6	21.5	31.4
Familial financial situation during childhood							
Difficult	32.4	20.6	26.5	51.0	42.4	49.1	31.2
Intermediate	59.5	72.2	61.2	43.2	49.5	42.1	55.3
Comfortable	8.1	7.1	12.3	5.8	8.2	8.8	13.5
Education							
0-7 years	44.7	30.6	32.0	45.2	35.4	36.4	26.4
8-12 years	42.7	45.8	45.6	38.2	37.1	45.6	37.8
13+ years	12.6	23.6	22.5	16.6	27.5	18.0	35.8
Marital status							
Married	76.2	84.6	80.4	78.0	82.6	57.6	65.1
Cigarette smoking							
Never	1.5	19.7	17.4	2.5	31.0	6.9	50.6
1-29 years	10.0	21.0	22.6	11.2	20.3	16.3	24.6
30+ years	88.4	59.3	60.0	83.2	50.8	76.1	24.8
Histology							
Squamous	41.9	--	--	35.4	--	19.6	--
Small cell	18.6	--	--	17.2	--	17.2	--
Adenocarcinoma	19.5	--	--	32.7	--	46.7	--
Other/unknown	20.1	--	--	14.8	--	16.6	--

The most commonly listed broad occupation groups for individuals exposed to cotton dust are listed in Table 2. They include: fabricating, assembling and repairing of textile, fur and leather products; fiber preparing, spinning, twisting, winding, reeling, weaving and knitting; apparel and furnishing service occupations, and; material recording, scheduling and distributing occupations. Not surprisingly, the most commonly listed industry was clothing and textile, followed by retail and wholesale trades. The specific occupational groups most commonly associated with cotton dust exposure were: tailors and dressmakers; patternmaking, marking and cutting of textile, fur and leather products; foremen in fabricating, assembling and repairing of textile, fur and leather products; sewing machine operators, textiles and similar materials; shipping and receiving clerks; pressing occupations; fabricating, assembling and repairing of textile, fur and leather products not elsewhere classified.

**Table 2:** **Most commonly listed broad occupation and industry groups for persons exposed to cotton dust and wool dust in two studies in Montreal, Canada, cases and controls combined** ^**a**^

	Study 1	Study 2
Cotton Dust	Total N exposed = 222	Total N exposed = 510
Occupation: n (%)	Fabricating, assembling and repairing occupations: textile, fur and leather products: 72 (32.4%)	Fabricating, assembling and repairing occupations: textile, fur and leather products: 232 (45.5%)
	Fiber preparing, spinning, twisting, winding, reeling, weaving and knitting: 30 (13.5%)	Apparel and furnishings service occupations: 43 (8.4%)
	Apparel and furnishings service occupations: 21 (9.4%)	Fiber preparing, spinning, twisting, winding, reeling, weaving and knitting: 36 (7.1%)
	Material recording, scheduling and distributing occupations: 37 (16.7%)	Material recording, scheduling and distributing occupations: 35 (6.9%)
Industry: n (%)	Clothing industry: 75 (33.4%)	Textile industries: 239 (46.9%)
	Textile industries: 81 (36.5%)	Wholesale trade: 36 (7.1%)
	Retail trades: 34 (15.3%)	Clothing industry: 26 (5.1%)
	Wholesale trade: 31 (14.0%)	
**Wool Dust**	**Total N exposed = 161**	**Total N exposed = 228**
Occupation: n (%)	Fabricating, assembling and repairing occupations: textile, fur and leather products: 70 (43.5%)	Fabricating, assembling and repairing occupations: textile, fur and leather products: 124 (54.4%)
	Apparel and furnishings service occupations: 19 (11.7%)	Apparel and furnishings service occupations: 35 (15.4%)
	Fiber preparing, spinning, twisting, winding, reeling, weaving and knitting: 17 (10.5%)	Fiber preparing, spinning, twisting, winding, reeling, weaving and knitting: 17 (7.5%)
	Material recording, scheduling, and distributing occupations: 23 (14.2%)	Material recording, scheduling, and distributing occupations: 15 (6.6%)
Industry: n (%)	Clothing industry: 39 (24.2%)	Textile industries: 150 (65.8%)
	Textile industries: 82 (50.9%)	Wholesale trade: 25 (11.0%)
	Retail trades: 41 (25.3%)	Clothing industry: 9 (4.0%)
	Wholesale trade: 29 (18.0%)	

^a^ Numbers and percentages based on persons ever holding a job with the given occupation/industry code, over total subjects with the given exposure. Percentages may total over 100, due to persons holding multiple jobs in different occupations and industries.

As assessed by our team of expert industrial hygienists, lifetime prevalence of exposure to cotton dust among male controls was about 8% in Study 1 and 13% in Study 2 (Table 3). Lifetime exposure prevalence was about 25% among female controls in Study 2. It seems that there was some shift in the threshold for assigning exposure between Study 1 and Study 2, since the increase among males was concentrated among assignments with the designation “possible” exposure and low concentration. Consequently, whereas cumulative cotton dust exposure was about evenly divided between substantial and non-substantial levels in Study 1, in Study 2 the majority of exposure was in the non-substantial category. Among those with cotton dust exposure, the majority was considered definitely exposed, and for at least 30% of their working hours (Table 3). About one-third had been exposed to cotton dust for 1-5 years, and 28% for >20 years. Exposure concentration was generally lower in Study 2 compared to Study 1. Exposure prevalence was somewhat lower for wool dust than for cotton dust, though the overall patterns were similar. As expected there was some overlap between these two textile exposures. In Study 1, out of 510 subjects exposed to cotton dust, 37.3% (n = 190) were also exposed to wool dust; in Study 2, 52.7% (n = 117) of 222 subjects exposed to cotton dust were also exposed to wool dust. Other exposures commonly assigned to jobs with cotton exposure were treated fibers, synthetic fibers, aliphatic aldehydes, formaldehyde, and magnetic and pulsed electromagnetic fields.

**Table 3:** **Frequency of different dimensions of exposure to cotton dust and wool dust in two studies in Montreal, Canada, cases and controls combined**

Exposure characteristic	Cotton Dust		Wool Dust	
	Study 1	Study 2	Study 1	Study 2
	(N=2739)	(N=2716)	(N=2739)	(N=2716)
	%	%	%	%
Level of exposure				
Non-substantial	4.1	17.2	2.5	7.4
Substantial	4.0	1.6	3.4	1.0
Exposure concentration^a^				
Low	2.6	15.9	1.6	6.9
Medium	4.2	2.4	3.8	1.4
High	1.3	0.5	0.5	0.2
Confidence level^a^				
Possible	0.1	2.0	0.04	1.1
Probable	1.3	5.1	1.1	1.9
Definite	6.7	11.7	4.8	5.5
Frequency ^a^				
<5% of work week	0.3	0.4	0.3	0.2
5-30% of work week	1.6	3.8	1.4	1.7
>30% of work week	6.2	14.4	4.3	6.5
Duration				
1-5 years	2.2	7.7	1.3	3.5
6-20 years	3.1	6.9	2.2	3.3
>20 years	2.8	4.2	2.4	1.6

^a^ Value is an average weighted by job duration, if reported for >1 job and/or time period.

Table 4 shows adjusted ORs between each exposure and lung cancer, and in each study. An OR was estimated with each control group in Study 1, for each sex in Study 2, and for a pooled analysis. We show results corresponding to ever exposure and to substantial exposure, as defined above. The pooled analysis indicates a weak effect (OR = 1.2) of borderline significance for any exposure (concentrated among males when compared with population controls), and non-statistically significant for substantial exposure. For wool dust, no significant excess risks were observed. Since the proportion of proxy respondents was higher among cases than among controls (29% and 38% of cases in Study 1 and 2, respectively, and 13% and 8% among controls), some differential misclassification of exposure might have occurred and resulted in biased OR estimates. We therefore repeated the analyses in Table 4, restricting to self-respondents only. The results were similar to those in the main analysis (OR for any exposure to cotton dust of 1.0, 95% CI: 0.8-1.2, and OR for substantial exposure to cotton dust of 1.2, 95% CI: 0.7-2.0). We also repeated the analyses, adjusting for smoking with the following three variables instead of the CSI: smoking status (ever/never), natural logarithm of cigarette-years, and years since cessation. Results did not differ from those presented in Table 4 (data not shown).

**Table 4:** **Odds ratios for association between cumulative exposure to cotton and wool dust, and lung cancer in two case-control studies in Montreal, Canada**

Exposure group	Study 1				Study 2				Pooled	
		Population controls	Cancer controls	All controls, weighted	Males		Females		Population controls	
	n^a^	OR^b^(95% CI)	OR (95% CI)	OR (95% CI)	n	OR (95% CI)	n	OR (95% CI)	n	OR (95% CI)
**Cotton dust**										
Ever exposure	66	1.4 (0.9-2.3)	1.0 (0.7-1.4)	1.2 (0.8-1.7)	108	1.4 (1.0-2.0)	131	1.0 (0.7-1.5)	305	1.2 (1.0-1.5)
Substantial exposure	30	1.5 (0.7-3.3)	0.8 (0.5-1.3)	1.0 (0.6-1.8)	14	1.1 (0.5-2.5)	5	1.0 (0.2-4.5)	49	1.2 (0.7-2.0)
**Wool dust**										
Ever exposure	42	1.2 (0.7-2.3)	0.8 (0.5-1.3)	1.0 (0.6-1.6)	46	0.9 (0.6-1.4)	47	1.2 (0.7-2.0)	135	1.0 (0.8-1.4)
Substantial exposure	22	1.3 (0.6-3.0)	0.7 (0.4-1.2)	0.9 (0.5-1.7)	8	1.5 (0.5-4.5)	6	7.6 (0.5-107.9)	36	1.5 (0.8-2.8)

^a^ n = number of exposed cases.

^b^ OR refers to odds ratio, adjusted for: age, ethnicity (French Canadian or other), years of education (0-7, 8-12 or 13+), familial financial situation during childhood (difficult, intermediate or comfortable), proxy respondent (yes or no), cumulative smoking index, and any occupational exposure to asbestos, chromium, nickel or silica. Pooled results are additionally adjusted for study.

We evaluated whether there was a difference in the effect of cotton dust exposure according to age at first exposure. Approximately two-thirds of exposed subjects had their first exposure before age 25, and we used this as the cut-point for a stratified analysis. Among those first exposed before age 25, the OR corresponding to ever exposure vs. never exposed was 1.2 (95% CI: 0.9-1.6) and that corresponding to substantial exposure was 1.1 (95% CI: 0.6-2.1). Analogous estimates for those first exposed at ages 25 and older were 1.6 (95% CI: 1.1-2.2) and 1.3 (95% CI: 0.5-3.0).

Table 5 shows results for each of the three major histologic subtypes of lung cancer. There were no statistically significant deviations from the null value for squamous cell or small cell carcinoma, but there was a significantly increased risk when restricting to adenocarcinoma cases (OR = 1.6, 95% CI: 1.2-2.2). Since some previous studies reported effect modification by smoking, we also analyzed the exposure-cancer associations separately in different smoking strata, namely in a category combining never smokers with light smokers and in another of medium to heavy smokers. As shown in Table 6, the association between ever exposure to cotton dust and lung cancer was slightly stronger in the stratum of medium-heavy smokers (OR = 1.3, 95% CI: 1.0-1.7), but there was no effect modification evident with ever exposure to wool dust.

**Table 5:** **Odds ratios for association between cotton and wool dust ever exposure and lung cancer in two studies in Montreal, stratified by histological type of lung cancer**

Exposure group	Study 1				Study 2				Pooled	
		Population controls	Cancer controls	All controls, weighted	Males		Females		Population controls	
	n^a^	OR^b^(95% CI)	OR (95% CI)	OR (95% CI)	n	OR (95% CI)	n	OR (95% CI)	n	OR (95% CI)
**Cotton dust**										
Squamous	23	1.2 (0.6-2.3)	0.8 (0.5-1.3)	1.0 (0.6-1.6)	37	1.3 (0.8-2.0)	19	0.9 (0.4-1.7)	79	1.1 (0.8-1.5)
Small cell	7	0.8 (0.3-2.2)	0.6 (0.2-1.0)	0.7 (0.3-1.5)	16	1.2 (0.6-2.5)	30	1.1 (0.5-2.3)	53	1.2 (0.8-1.8)
Adenocarcinoma	21	2.7 (1.4-5.3)	1.7 (1.0-2.9)	2.1 (1.2-3.7)	46	1.9 (1.2-3.0)	61	1.2 (0.7-1.8)	128	1.6 (1.2-2.2)
**Wool dust**										
Squamous	14	1.1 (0.5-2.4)	0.6 (0.3-1.2)	0.8 (0.4-1.6)	13	0.6 (0.3-1.3)	6	0.7 (0.3-2.2)	33	0.8 (0.5-1.2)
Small cell	7	1.1 (0.4-3.2)	0.8 (0.4-1.9)	1.0 (0.4-2.3)	6	0.8 (0.3-2.0)	7	0.8 (0.3-2.6)	20	0.9 (0.5-1.6)
Adenocarcinoma	12	2.2 (1.0-4.9)	1.3 (0.7-2.5)	1.6 (0.8-3.2)	22	1.4 (0.8-2.5)	24	1.2 (0.6-2.4)	58	1.4 (1.0-2.0)

^a^ n = number of exposed cases.

^b^ OR refers to odds ratio, adjusted for: age, ethnicity (French Canadian or other), years of education (0-7, 8-12 or 13+), familial financial situation during childhood (difficult, intermediate or comfortable), proxy respondent (yes or no), cumulative smoking index (CSI), and any occupational exposure to asbestos, chromium, nickel or silica. Pooled results are additionally adjusted for study.

**Table 6:** **Odds ratios for association between cotton and wool dust ever exposure and lung cancer in two studies in Montreal, stratified by smoking status**

Exposure group	Study 1				Study 2				Pooled	
		Population controls	Cancer controls	All controls, weighted	Males		Females		Population controls	
	n^a^	OR^b^(95% CI)	OR (95% CI)	OR (95% CI)	n	OR (95% CI)	n	OR (95% CI)	n	OR (95% CI)
**Cotton dust**										
All subjects	66	1.4 (0.9-2.3)	1.0 (0.7-1.4)	1.2 (0.8-1.7)	108	1.4 (1.0-2.0)	131	1.0 (0.7-1.5)	305	1.2 (1.0-1.5)
Never/Low smokers^c^	8	0.7 (0.3-2.0)	0.7 (0.3-1.6)	0.7 (0.3-1.6)	15	2.1 (1.1-4.2)	12	0.9 (0.4-1.9)	37	1.0 (0.7-1.6)
Medium/High Smokers^c^	58	2.1 (1.1-4.0)	1.1 (0.7-1.6)	1.4 (0.9-2.2)	91	1.3 (0.8-1.8)	119	1.0 (0.6-1.6)	266	1.3 (1.0-1.7)
**Wool dust**										
All subjects	42	1.2 (0.7-2.3)	0.8 (0.5-1.3)	1.0 (0.6-1.6)	46	0.9 (0.6-1.4)	47	1.2 (0.7-2.0)	135	1.0 (0.8-1.4)
Never/Low smokers^c^	4	1.0 (0.3-3.3)	0.6 (0.2-1.8)	0.7 (0.2-2.1)	5	1.1 (0.4-3.0)	6	1.2 (0.4-3.1)	15	0.9 (0.5-1.7)
Medium/HighSmokers^c^	38	1.5 (0.7-3.2)	0.9 (0.6-1.4)	1.1 (0.6-1.9)	40	0.9 (0.5-1.4)	41	1.2 (0.6-2.4)	119	1.0 (0.7-1.5)

^a^ n = number of exposed cases.

^b^ OR refers to odds ratio, adjusted for: age, ethnicity (French Canadian or other), years of education (0-7, 8-12 or 13+), familial financial situation during childhood (difficult, intermediate or comfortable), proxy respondent (yes or no), cumulative smoking index (CSI), and any occupational exposure to asbestos, chromium, nickel or silica. Pooled results are additionally adjusted for study.

^c^ Low smokers are defined as those having a CSI value ≤25% percentile of CSI values among ever smoker.

Some previous studies were based on cohorts in certain high exposure industries or occupations, whereas our database included workers across the entire spectrum of occupations and industries. To determine whether exposure to cotton dust in different occupations or industries is associated with different risks, we carried out analyses of cotton dust exposure, stratified on the main industries in which cotton dust exposure occurred in our population. Due to small numbers, these subgroup analyses produced rather unstable risk estimates, but there was no evidence of a protective effect of cotton dust exposure within any industry (data not shown).

## Discussion

We used data from two large case-control studies conducted in Montreal to assess the relationship between occupational exposure to cotton dust and wool dust and risk of lung cancer. Subjects in Study 1 were in their active work years roughly from the 1940s to the 1970s, whereas the active period for Study 2 subjects was the 1950s to 1980s. Thus there was considerable overlap. It is likely that the average concentrations of exposure declined between the two studies because of improved industrial hygiene and use of personal protective equipment. Historically the Province of Quebec was the hub of the clothing and textile industries in Canada, and despite decreasing quotas and increasing offshore production, it so remains with approximately 50,000 workers employed in these fields [33]. Lifetime prevalence of exposure was higher in Study 2 than in Study 1 because females, who were disproportionately active in the textile and clothing industries, were not included in Study 1, and because there seemed to be a lower threshold among our exposure experts for assigning these exposures in Study 2 than in Study 1. These various trends between the two studies did not bias our risk estimates which were stratified by study and adjusted for study in the pooled analyses.

Overall there was little evidence of a protective effect of cotton dust exposure on lung cancer, in Study 1 or Study 2, in males or in females. In fact the point estimates were usually slightly above 1.0 and attained borderline statistical significance in some of the contrasts. Nor do the analyses by histologic type provide clear evidence of protective effects of cotton dust; indeed the strongest association indicated an excess risk of adenocarcinoma of the lung. Our results for wool dust, which overlaps with exposure to cotton dust, tended to be close to the null value, except in small and statistically unstable subgroups.

While most studies of cotton textile workers have reported protective effects, and a meta-analysis estimated a summary decrease in risk of 28%, several studies have either found no association between work in the textile industry and lung cancer risk [14-16], or a suggestion of increased risk of lung cancer [34]. Our results on cotton dust and wool dust were closer to the null than to a protective effect. Most previous studies of cotton exposed workers had no or little information available on smoking habits. The most prominent exception was the study of Shanghai female textile workers, which collected smoking information from all subjects, and in which there were very few smokers [6]. The validity of the smoking data is questionable since the relative risk estimates for smoking and lung cancer were quite low compared with other studies which have estimated relative risks among female smokers. However, very low cumulative smoking might explain this weak association. In any case, after adjusting for smoking, the investigators reported a strong protective effect of cotton dust. More recent studies suggested an increased risk of lung cancer among workers exposed to organic dust [35]. In addition, further analyses of the Shanghai female textile workers suggested increased lung cancer risk among those whose first exposure to endotoxin occurred in the more distant past, and thus at a younger age [36,37]. In contrast, we did not find evidence of a stronger effect among those first exposed at a young age.

The failure of our study to demonstrate a protective effect of cotton dust exposure is unlikely to be due to simple measurement error in the assessment of cotton dust exposure, as this is not an exposure that is particularly difficult for experts to identify in a work history, given the information that was available to our experts (industry, occupation, worker’s tasks, and other details of the workplace). However, if there really is a protective effect of cotton dust exposure, we may have failed to find such an association for one of the following reasons.

First, it may be that the intensity of exposure, on average, in our subjects was much less than that in the cohort studies that have previously reported protective effects. Since ours was a population-based case-control study with workers exposed to cotton dust across a wide range of occupations and industries, the proportion of very highly exposed workers may have been low. Without absolute exposure measures it is hard to evaluate this possibility. Nevertheless, we can affirm that in our population-based study covering the range of exposure intensities, there was no meaningful departure from the null. Second, there may be an effect modification by smoking. The strongest evidence of a protective effect of cotton dust comes from studies conducted in China where there were few smokers [6]. In our study, there are too few nonsmokers to be able to affirm whether or not there is a protective effect in this stratum. The third possible reason for our failure to detect a protective effect has to do with the “endotoxin hypothesis” [18,19]. If there is indeed a protective effect due to endotoxin content of cotton dust, then cotton dust with less endotoxin content may not be protective. Marchand et al have reported on endotoxin measurements taken in four Quebec textile mills [38]. They found measureable and even quite high levels throughout the plants, with considerable variability in concentration by plant, process, work station, and season. While the lack of standardized analytical method prevents the direct comparison of Marchand et al’s results to a slightly older study also performed in textiles mills in Taiwan [39], the concentrations in both studies were of the same order of magnitude, reaching > 500 ng of endotoxins per cubic meter in the most exposed areas.

While some of our exposed subjects were from textile mills, most were from occupations and industries further down the production and retailing chain of textile products. Unfortunately there is little hard data available on endotoxin content of cotton dust or on ambient endotoxin exposure levels in such environments. The evidence from the textile mills remains ambiguous, suggesting lower levels as one goes further in the processing chain within the mill [39], but also elevated levels in later processing steps such as spinning and winding [38]. We presume that the processing of cotton fibers leads to reduction of endotoxin content and that exposure to endotoxins would be much lower further down in the retailing chain of textile products. Thus, while our results are informative about cotton and wool dust in relation to lung cancer, without additional data on endotoxin levels in a wider range of cotton-exposed occupations, it is difficult to assess whether our results are informative about endotoxins and lung cancer. The only hint from our own data was that in analyses of subgroups exposed to cotton dust in different occupations, we saw no difference in the OR estimates according to the occupation in which the exposure to cotton dust occurred (e.g., occupation codes indicating fiber preparation vs. occupation codes indicating textile product fabrication). But these were based on small numbers with wide confidence intervals.

In assessing the associations between cotton and wool dusts and lung cancer, our study had several strengths, including: large sample sizes with fairly high numbers of exposed cases and controls; fairly high participation rates which reduces the risk of selection bias; complete lifetime work histories with detailed descriptions of each job; job-by-job evaluation of exposures by a team of experts; detailed lifetime history of smoking; and information on a host of other covariates. While there were large numbers of proxy respondents, the results of analyses restricted to self-respondents were virtually identical to the main ones. Notwithstanding these strengths, the study was limited by lack of measurements of cotton and wool dust, and inferences regarding endotoxins are limited by lack of endotoxin measurements.

## Conclusion

In conclusion, neither cotton dust nor wool dust showed associations with lung cancer. We found no evidence for a decreased risk of lung cancer among persons exposed to cotton dust.
